# Poly(Neutral Red)–Silver Nanorods–Carbon Nanotubes Composite-Based Ratiometric Electrochemical Sensor for Rapid Detection of Histamine in Crayfish

**DOI:** 10.3390/foods15162917

**Published:** 2026-08-20

**Authors:** Shuo Duan, Chunyan Liao, Huang Dai, Yunhan Liu, Yongjiang Zhang, Qiao Wang, Zhanming Li

**Affiliations:** 1 Department of Environment and Quality Testing, Chongqing Chemical Industry Vocational College, Chongqing 400020, China; 18623415305@163.com (C.L.); liuyunhan0914@163.com (Y.L.); yjzhang008@163.com (Y.Z.); wangqiao8577@163.com (Q.W.); 2 College of Food Science and Engineering, Wuhan Polytechnic University, Wuhan 430024, China; 3 School of Grain Science and Technology, Jiangsu University of Science and Technology, Zhenjiang 212000, China; lizhanming@just.edu.cn

**Keywords:** histamine, ratiometric electrochemical sensor, silver nanorods, carbon nanotubes, poly(neutral red), crayfish, freshness detection

## Abstract

Histamine accumulation in spoiled aquatic products poses significant food safety risks, yet rapid and accurate on-site detection remains challenging due to the lack of direct electrochemical activity of histamine and severe matrix interference in complex biological samples. Herein, we report a ratiometric electrochemical sensor based on a poly(neutral red)–silver nanorods–carbon nanotubes (PNR-AgNRs/CNTs) composite for rapid histamine detection in crayfish. The sensor leverages a dual-signal strategy wherein histamine competitively suppresses the PNR electrochemical signal while leaving the AgNRs signal unaffected, enabling self-referencing quantification via the I_PNR_/I_AgNRs_ ratio. This design effectively eliminates interference from instrument drift and sample matrix. The sensor exhibited a linear response range of 1–150 μmol/L with a detection limit of 0.16 μmol/L, along with excellent stability and reproducibility. When applied to crayfish samples, the method achieved satisfactory recoveries (95.9–102.3%) and showed no significant difference compared to the national standard method. By circumventing the need for histamine’s direct electrochemical response, this ratiometric approach provides a robust platform for rapid on-site assessment of aquatic product freshness.

## 1. Introduction

Aquatic products represent a crucial component of the global food supply chain, providing abundant high-quality proteins, polyunsaturated fatty acids, vitamins, and minerals that contribute to dietary optimization and alleviation of food shortages [1]. The global aquatic industry has achieved an annual production value exceeding 400 billion USD, creating over 60 million employment opportunities in coastal countries and regions. China, as the world’s largest producer and consumer of aquatic products, reported a total output value of the aquatic industry surpassing 1.3 trillion RMB in 2023, with aquaculture production ranking first globally for 33 consecutive years [2]. Crayfish (Procambarus clarkii), as an important economic aquatic species in China, reached a total production of 2.89 million tons in 2022, with a comprehensive output value exceeding 458 billion RMB, encompassing complete industrial chains from ecological aquaculture and cold chain transportation to deep food processing and catering services [3].

However, aquatic products are highly susceptible to microbial contamination and enzymatic spoilage during harvesting, storage, transportation, and processing due to their high moisture content, rich protein composition, and active endogenous enzymes. This spoilage not only leads to nutrient loss and degradation of eating quality but also generates harmful substances, posing food safety risks to consumers and causing severe economic losses to enterprises [4,5]. Therefore, establishing scientific and reliable freshness evaluation methods for aquatic products is of great significance for ensuring food safety, protecting consumer rights, and promoting high-quality industrial development.

Freshness is a critical indicator for evaluating the quality of aquatic products, directly influencing sensory characteristics, nutritional composition, and processing performance. During the spoilage process, adenosine triphosphate (ATP) in aquatic products undergoes stepwise decomposition under enzymatic action, successively producing purine compounds such as hypoxanthine (Hx) and xanthine (Xa); histidine is decarboxylated by microbial histidine decarboxylase to generate histamine; and trimethylamine oxide is reduced to trimethylamine (TMA) and other volatile basic nitrogen compounds under microbial action [6,7,8]. Consequently, metabolites including xanthine, hypoxanthine, histamine, and trimethylamine exhibit significant correlations with the degree of spoilage and can serve as characteristic freshness biomarkers. Among these, histamine, as one of the most toxic biogenic amines, is particularly important for assessing the freshness and safety of aquatic products [9].

Histamine, chemically known as 2-(4-imidazolyl)ethylamine, is a common bioactive amine widely distributed in seafood, fermented foods, pickled products, and certain beverages [10]. During food spoilage, histamine is produced from histidine in proteins catalyzed by microbial histidine decarboxylase, with its generation significantly influenced by storage temperature and pH conditions [11]. As a potent foodborne toxin, high-dose histamine exposure can cause severe histamine poisoning. Concentrations of 8–40 ppm may induce mild food poisoning, while levels exceeding 100 ppm can lead to headache, nausea, vomiting, migraine, palpitations, blood pressure fluctuations, and localized allergic reactions; in extreme cases, which may be life-threatening [12,13]. The European Union food safety standard stipulates that histamine content in fresh fish must not exceed 200 mg/kg, and fish products are deemed to be spoiled when histamine concentration reaches 50 mg/kg (approximately 450 μmol/L), with concentrations exceeding 500 mg/kg (approximately 4500 μmol/L) posing potentially fatal risks [14]. China’s national standard GB 2733-2015 [15] specifies that histamine content in fresh and frozen aquatic products must not exceed 40 mg/kg [16]. Therefore, rapid and accurate detection of histamine is essential for ensuring food quality and protecting consumer health.

Currently, histamine detection methods mainly include high-performance liquid chromatography (HPLC), gas chromatography (GC), fluorescence spectroscopy, molecular imprinting technology, and enzyme-linked immunosorbent assay (ELISA) [17,18,19,20]. Although these methods offer high detection precision, they generally suffer from expensive instrumentation, tedious sample pretreatment, requirement for highly skilled operators, and long detection cycles, making them unsuitable for on-site rapid screening and real-time monitoring. In recent years, electrochemical sensing technology has attracted widespread attention for food safety detection due to its advantages of simple operation, low cost, rapid response, and high sensitivity [21,22].

Electrochemical sensors can significantly enhance electron transfer rate and catalytic activity through electrode surface modification with nanomaterials, enabling highly sensitive detection of target analytes [23,24]. However, histamine molecules inherently lack direct electrochemical activity and cannot be directly detected by electrochemical methods. Furthermore, aquatic products represent complex biological samples with severe matrix interference, which constitutes a critical bottleneck restricting the application of electrochemical detection for histamine in aquatic products [22].

Ratiometric electrochemical sensors, which employ dual-signal output mechanisms, can effectively eliminate or reduce interference from environmental factors such as temperature, pH, and ionic strength, as well as instrument fluctuations, thereby significantly improving the accuracy and reliability of detection results [24,25,26]. Compared with traditional single-signal sensors, ratiometric sensors introduce an internal reference signal that can effectively eliminate errors arising from differences in electrode modification, instrumental fluctuations, and variations in environmental conditions, while also reducing sample matrix interference in complex matrices such as biological and food samples [26].

Rationale for sensor design. The selection of poly(neutral red) (PNR) as the recognition element is grounded in established literature demonstrating that protonated PNR undergoes phosphate-mediated ion-pair formation with multiply protonated biogenic amines, which restricts polymer doping/de-doping dynamics and produces a quantifiable attenuation of the polymer redox current [27]. Kumar et al. have validated this mechanism for cadaverine and putrescine detection, showing that PNR can be grown in situ on an electrode surface via electrochemical polymerization, possesses intrinsic redox activity, and interacts competitively with biogenic amines through ion-pairing—features that make it highly attractive for amine sensing applications. Similarly, our group has also reported a carbon nanotube–poly(neutral red) electrochemical competitive sensor for putrescine detection in Procambarus clarkii, which relied on the same competitive interaction between PNR and biogenic amines [28]. However, the previous sensor reported in that study relied on a single PNR signal, leaving it susceptible to matrix interference and instrumental drift [27,28]. To overcome this limitation, a ratiometric dual-signal strategy was deemed necessary, which requires an additional electrochemically active component that provides a stable, potential-resolved internal reference signal. Silver nanostructures were chosen for this role based on our previous work [29,30] which demonstrated that silver nanoparticles exhibit well-defined and reproducible electrochemical oxidation signals, making them suitable as signal sources in electrochemical sensing platforms. Specifically, silver nanorods (AgNRs) display a distinct redox couple at approximately 0.1 V and −0.6 V that is electrochemically orthogonal to the PNR signal (about −0.41 V), enabling baseline-resolved dual-signal acquisition. Carbon nanotubes (CNTs) were employed as a three-dimensional scaffold to support and disperse AgNRs, leveraging their large specific surface area and excellent electrical conductivity to facilitate electron transfer. In this architecture, the AgNRs signal functions as an intrinsic internal reference that remains insensitive to biogenic amine binding, whereas the PNR signal serves as the analyte-responsive probe, thereby achieving self-referencing quantification without requiring exhaustive screening of alternative polymers or noble metals.

**Scheme 1:**
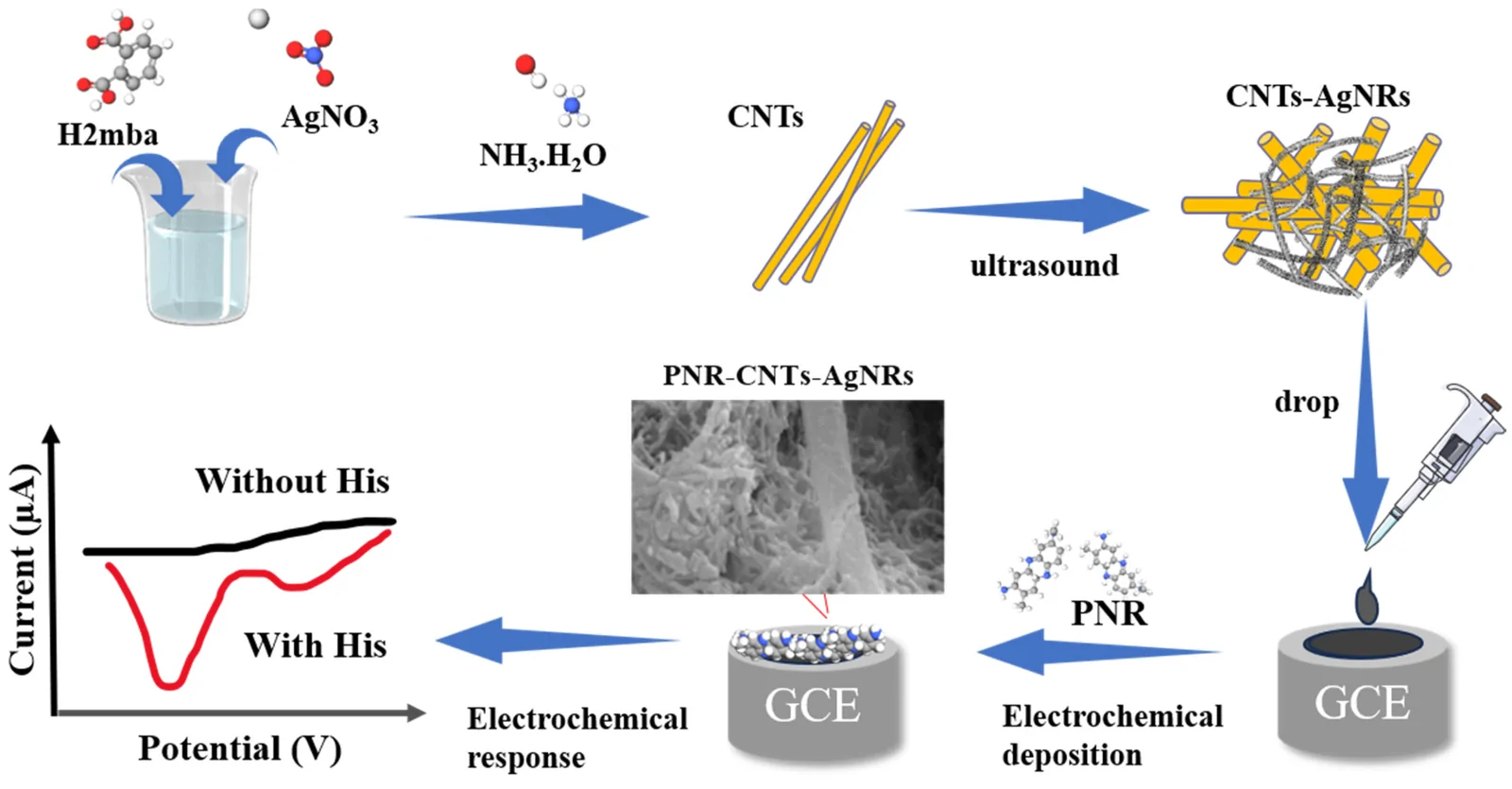
Brief schematic illustration of the electrode preparation and electrochemical detection of histamine.

In this study, we prepared the carbon nanotubes (CNTs)–silver nanorods (AgNRs)–poly-neutral red (PNR) composite material and constructed a novel ratiometric electrochemical sensor to address the limitations of existing rapid detection technologies for histamine in aquatic products. Since histamine molecules suppress the electrochemical signal of PNR on the electrode without affecting the AgNRs signal, changes in the ratio of the two electrochemical signals enable rapid electrochemical detection of histamine concentration (Scheme 1). The ratiometric detection strategy effectively excluded matrix interference from biological samples, and achieved accurate detection of histamine under complex matrix conditions in aquatic products.

## 2. Materials and Methods

### 2.1. Reagents and Materials

All the reagents and materials used in this research were fully detailed in the Supplementary Materials Section S1.

### 2.2. Instrumentation

All the instrumentation used in this research was fully detailed in the Supplementary Materials Section S1.

### 2.3. Synthesis of Silver Nanorods (AgNRs)

AgNRs were synthesized via a liquid-phase reduction method with optimization based on literature procedures [31]. The detailed experimental procedures are presented in the Supplementary Materials Section S1.

### 2.4. Fabrication of PNR-AgNRs-CNTs/GCE

The detailed experimental procedures for PNR-AgNRs-CNTs/GCE preparation are presented in the Supplementary Materials Section S1.

### 2.5. Sample Pretreatment

Crayfish samples were purchased from local markets. The pretreatment procedure was performed in the Supplementary Materials Section S1.

### 2.6. Electrochemical Measurements

All electrochemical measurements were conducted in 0.1 M phosphate buffered saline (PBS, the pH value is 6.0) at room temperature. The detailed electrochemical techniques employed in this work are presented in the Supplementary Materials Section S1.

### 2.7. National Standard Method Validation

To validate the accuracy of the proposed electrochemical method, the national standard method (GB 5009.208-2016) [32] was employed as a reference. The detailed experimental procedures are presented in the Supplementary Materials Section S1.

### 2.8. Data Analysis

The recovery and relative standard deviation (RSD) were calculated according to the following equations:

Recovery (%) = (C_detected_ − C_background_)/C_spiked_ × 100%

RSD (%) = SD/Mean × 100%

Prior to spiking experiments, the native histamine concentration (C_background_) in each untreated crayfish homogenate was determined independently using the proposed electrochemical sensor and the standard method (GB 5009.208-2016). All measurements were performed at least in triplicate, and data were expressed as mean ± standard deviation.

## 3. Results and Discussion

### 3.1. Characterization of PNR-AgNRs-CNTs Composite

**Figure 1:**
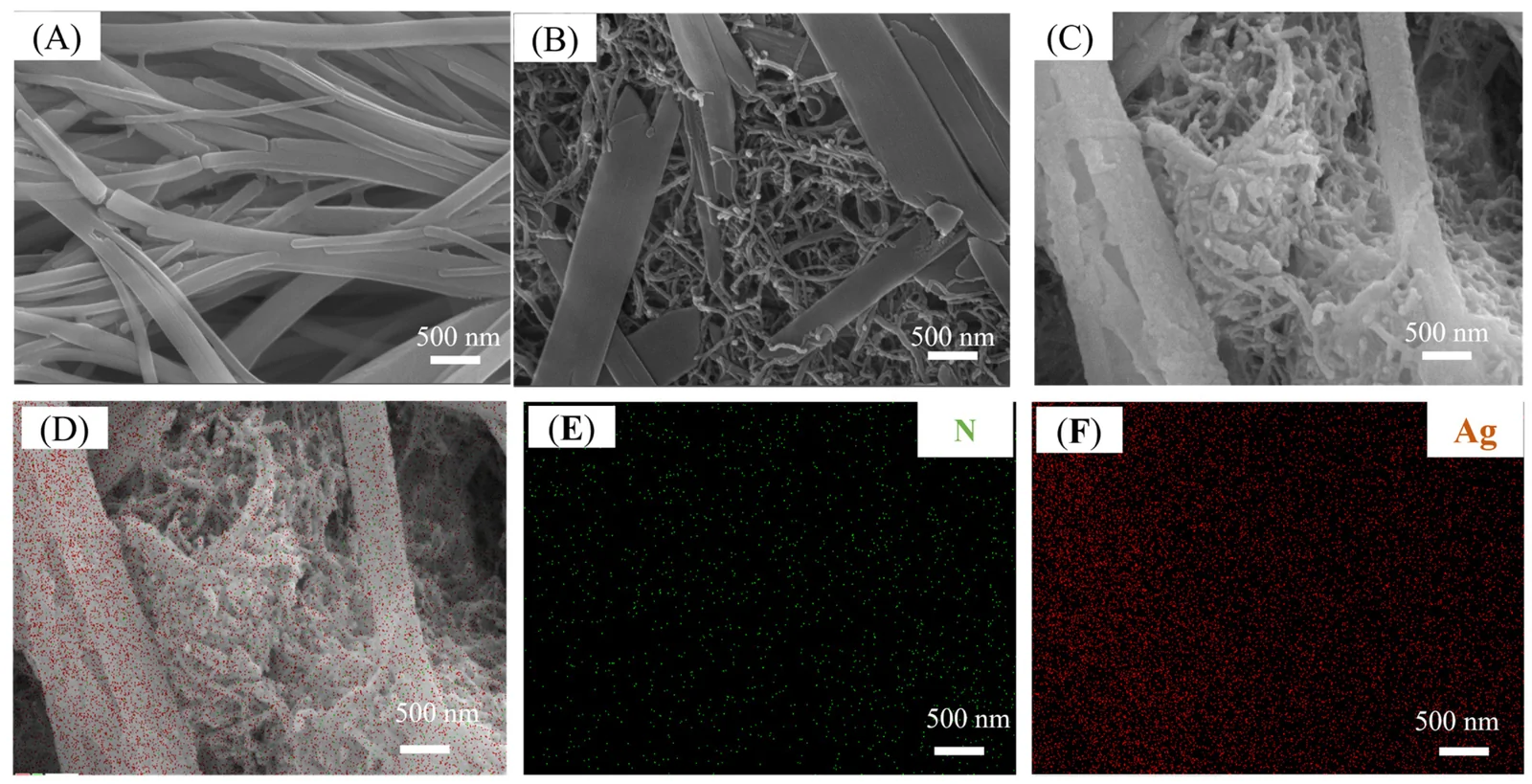
SEM micrographs showing the surface morphologies of (**A**) AgNRs/GCE, (**B**) AgNRs-CNTs/GCE, and (**C**) PNR-AgNRs-CNTs/GCE, revealing that the three-dimensional interlaced network is retained after PNR polymerization. (**D**–**F**) EDS elemental mapping of PNR-AgNRs-CNTs: (**D**) merged overlay, (**E**) nitrogen, and (**F**) silver, confirming uniform distribution of both elements across the composite surface. Accelerating voltage: 15 kV.

The surface morphologies of AgNRs/GCE, AgNRs-CNTs/GCE, and PNR-AgNRs-CNTs/GCE were characterized by SEM. As shown in Figure 1A, AgNRs exhibited distinct rod-like structures with lengths of approximately 200–500 nm and diameters of approximately 50–100 nm, relatively uniformly distributed on the GCE surface. After being composited with CNTs (Figure 1B), the AgNRs-CNTs displayed a three-dimensional interlaced network structure where thread-like CNTs and rod-like AgNRs were interconnected, forming a dense conductive network favorable for electron transfer. Following PNR polymerization (Figure 1C), the PNR-AgNRs-CNTs/GCE retained the three-dimensional interlaced structure, indicating that the polymerization process did not disrupt the composite architecture. EDS elemental mapping (Figure 1D–F) revealed uniform distribution of nitrogen (N) and silver (Ag) across the composite surface, confirming the successful synthesis of the PNR-AgNRs-CNTs composite.

**Figure 2:**
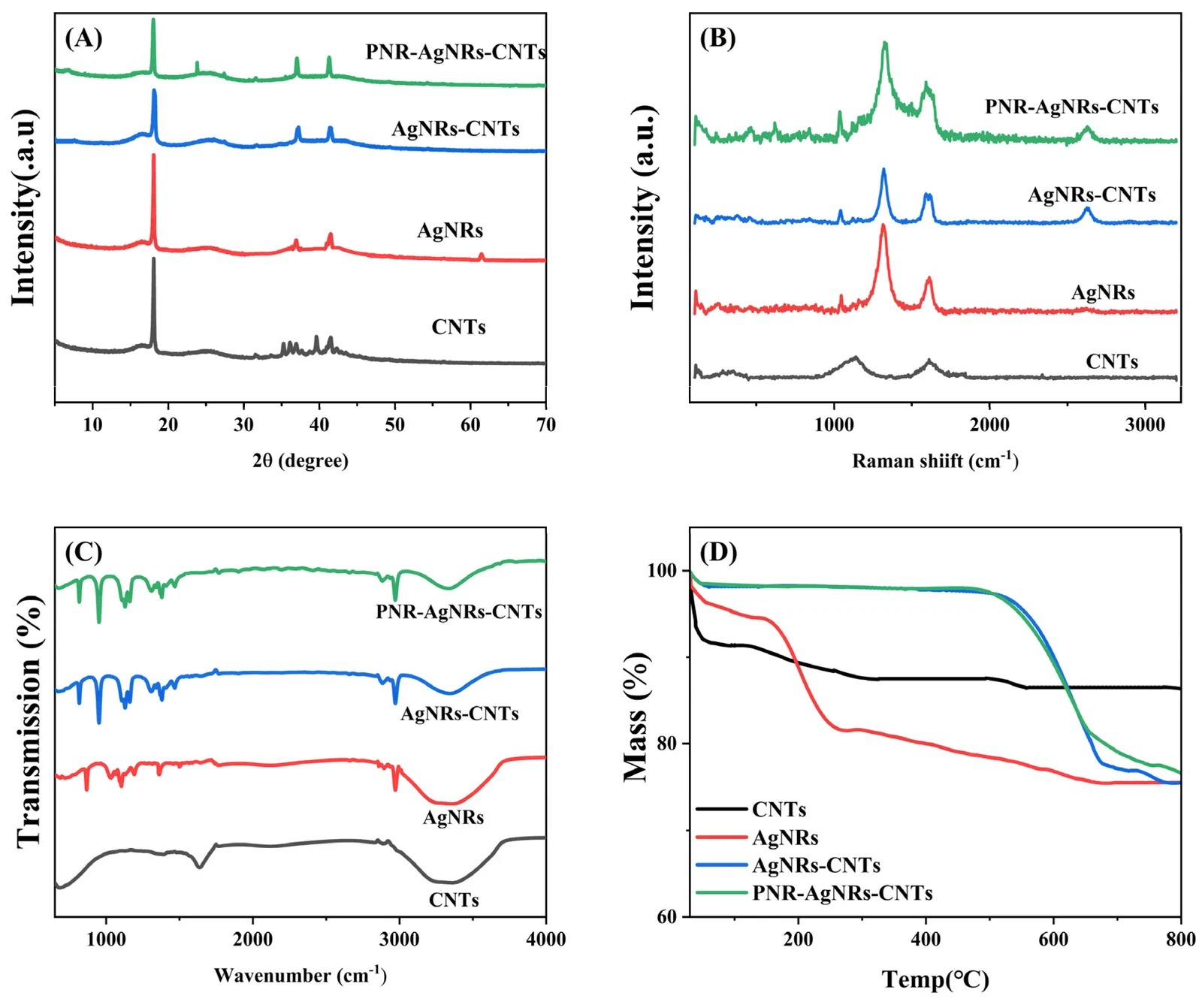
Physicochemical characterization of the synthesized materials. (**A**) XRD patterns (2θ = 20–80°, scan rate 5°/min) of CNTs (black), AgNRs (red), AgNRs-CNTs (blue), and PNR-AgNRs-CNTs (green); peaks at 38.0°, 41.5°, and 61.4° correspond to the (111), (200), and (220) planes of face-centered cubic silver (JCPDS No. 04-0783). (**B**) Raman spectra (532 nm excitation, 10 mW) showing the D and G bands of CNTs. (**C**) FTIR spectra (4000–400 cm^−1^, resolution 4 cm^−1^) confirming successful PNR coating. (**D**) TGA curves (N_2_ atmosphere, 10 °C/min) demonstrating that PNR encapsulation improves the thermal stability of the composite.

The XRD patterns of AgNRs, CNTs, AgNRs-CNTs, and PNR-AgNRs-CNTs are presented in Figure 2A. AgNRs and the composite materials exhibited three characteristic diffraction peaks at 38.0°, 41.5°, and 61.4°, corresponding to the (111), (200), and (220) crystal planes of face-centered cubic silver (JCPDS No. 04-0783), respectively, confirming the successful synthesis of AgNRs [31,33]. CNTs showed a characteristic carbon (002) peak near 26°. The AgNRs-CNTs composite displayed both silver and carbon characteristic peaks, indicating successful integration of the two materials. The XRD pattern of PNR-AgNRs-CNTs was similar to that of AgNRs-CNTs, with slightly reduced peak intensity, suggesting that PNR coating had minimal impact on the crystal structure.

As shown in Figure 2B, CNTs exhibited prominent carbon characteristic peaks at 1345 cm^−1^ (D band, corresponding to disordered carbon structure) and 1575 cm^−1^ (G band, corresponding to graphitic carbon structure). AgNRs displayed characteristic silver peaks in the 200–400 cm^−1^ range. The AgNRs-CNTs composite showed both carbon and silver characteristic peaks, confirming the coexistence of both components. The PNR-AgNRs-CNTs spectrum retained the characteristic peaks of the composite without significant new peaks, indicating that PNR coating did not alter the essential structure of the material.

The FTIR spectra (Figure 2C) showed that CNTs exhibited characteristic peaks at 3400 cm^−1^ (O–H stretching vibration) and 1630 cm^−1^ (C=C stretching vibration). In the AgNRs-CNTs composite, the presence of AgNRs enhanced the intensity of CNTs characteristic peaks, indicating interaction between AgNRs and CNTs. PNR-AgNRs-CNTs displayed characteristic absorption peaks of poly(neutral red) in the 1500–1600 cm^−1^ range, confirming successful PNR coating. The thermogravimetric curves (Figure 2D) revealed that CNTs began significant weight loss above 600 °C, while AgNRs exhibited excellent thermal stability with minimal weight loss. The AgNRs-CNTs composite showed intermediate weight loss behavior. Notably, PNR-AgNRs-CNTs exhibited a slightly higher initial decomposition temperature and reduced weight loss rate at high temperatures, indicating that the PNR coating improved the thermal stability of the composite by mitigating the high-temperature decomposition of silver and carbon components.

### 3.2. Electrochemical Performance Evaluation

**Figure 3:**
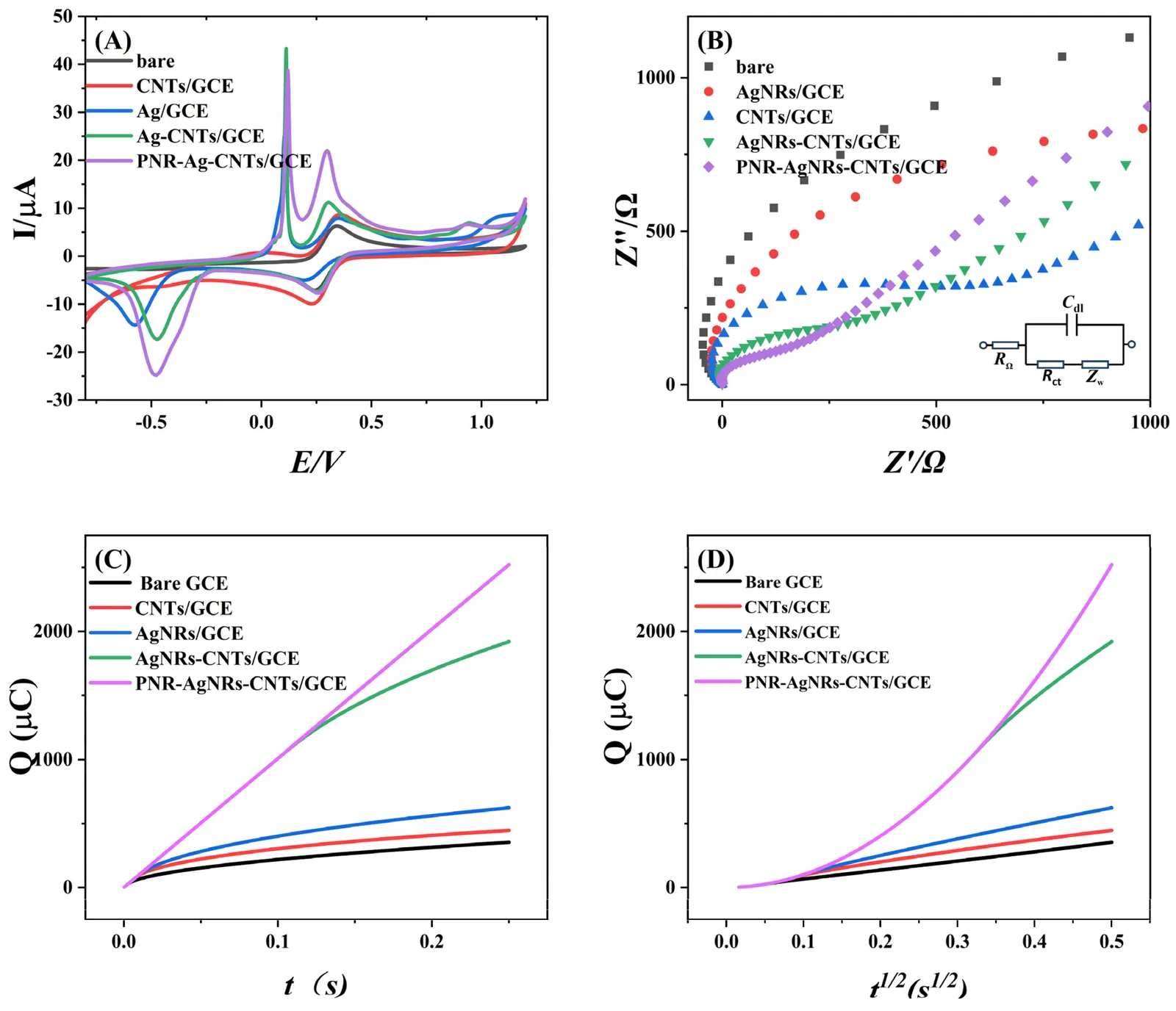
The CVs (**A**), EIS results (**B**), Q-t curves (**C**) and Q-t^1/2^ curves (**D**) for the bare GCE (black line), CNTs/GCE (the red line), AgNRs/GCE (the blue line), AgNRs-CNTs/GCE (the green line) and the obtained PNR-AgNRs-CNTs/GCE (the pink line). All the measurements were performed in 5 mmol/L K_3_[Fe(CN)_6_]/0.1 M KCl solution as the electrolyte, detailed conditions were listed in Section 2.6.

CV and EIS analyses: The electrochemical performance of different electrodes was evaluated in 5 mmol/L K_3_[Fe(CN)_6_]/0.1 M KCl solution. As shown in Figure 3A, the PNR-AgNRs-CNTs/GCE exhibited the highest redox peak currents among all tested electrodes, indicating optimal electrochemical performance. It is worth noting that the Ag-CNTs electrode exhibited superior electrochemical performance compared to the Ag electrode and the CNTs electrode. This is attributed to the conductive cross-linking structure formed between CNTs and Ag, which effectively promotes electron transfer at the electrode surface. Electrodes containing AgNRs (AgNRs/GCE, AgNRs-CNTs/GCE, and PNR-AgNRs-CNTs/GCE) displayed distinct silver oxidation-reduction peaks at approximately 0.1 V and −0.6 V, confirming successful loading of AgNRs, whose prominent electrochemical signal is suitable for ratiometric sensor construction. The EIS Nyquist plots (Figure 3B) demonstrated that PNR-AgNRs-CNTs/GCE exhibited the smallest semicircle diameter, corresponding to the lowest charge transfer resistance (R_ct_) and best conductivity among all electrodes.

Chronocoulometry (CC) analysis: CC measurement was employed to determine the electrochemically active surface area based on the Anson equation. As shown in Figure 3C,D, the PNR-AgNRs-CNTs/GCE exhibited the largest slope in the Q-t^1/2^ plot curve, indicating the largest electrochemically active surface area, which was approximately five-fold greater than that of bare GCE. This significant enhancement in active surface area contributes to the superior electrochemical performance of the composite electrode.

These results collectively demonstrate that the PNR-AgNRs-CNTs/GCE possesses excellent electrochemical performance and generates dual current signals from PNR and AgNR, fulfilling the fundamental requirements for a ratiometric electrochemical sensor.

### 3.3. Ratiometric Detection of Histamine

**Figure 4:**
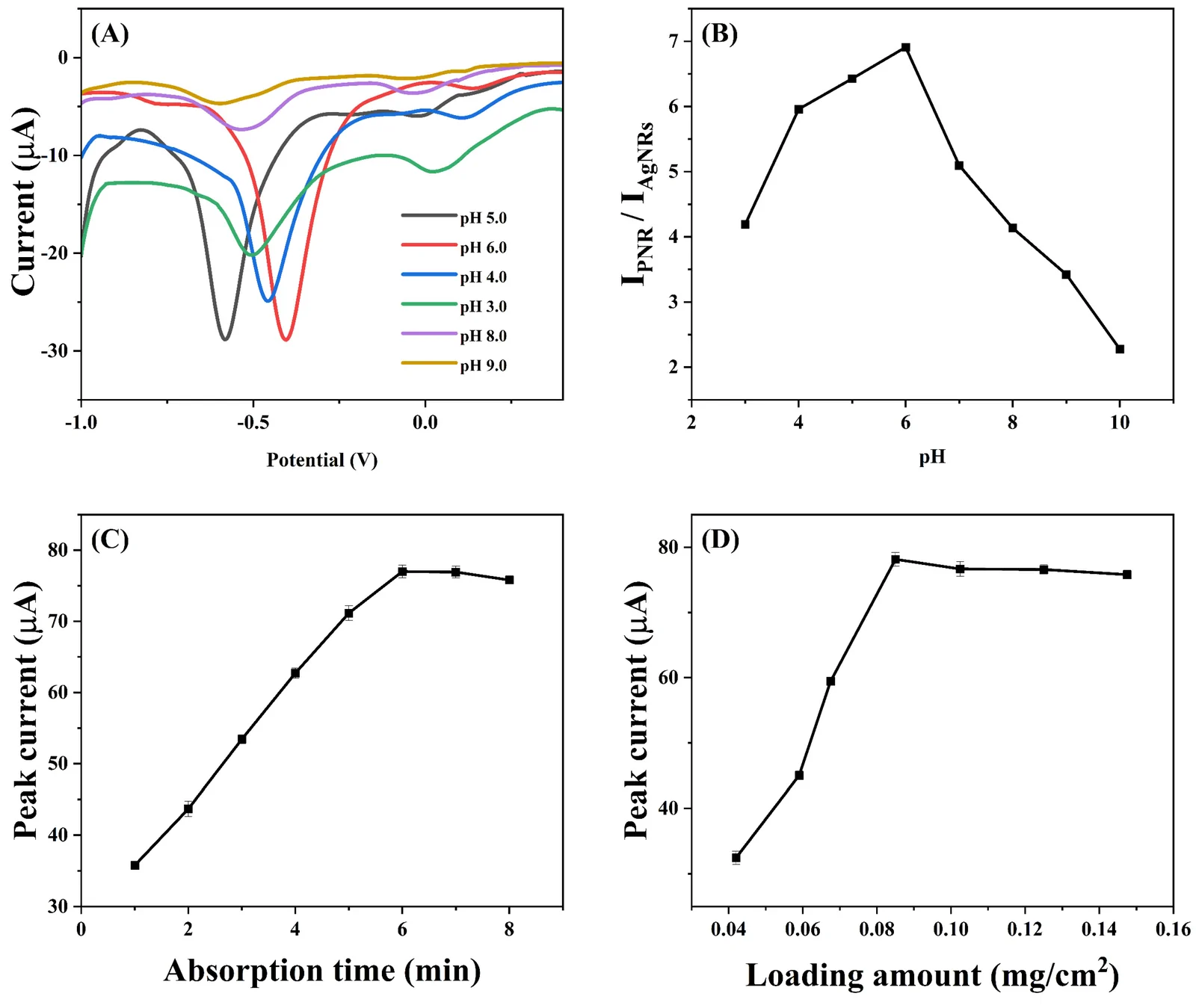
The DPVs for histamine solution at PNR-AgNRs-CNTs/GCE with different electrolyte pH values (**A**); the optimization results for pH value (**B**), absorption time (**C**) and loading amount of CNTs (**D**). All the tests were carried in the PBS electrolyte containing 150 μmol/L histamine.

**Figure 5:**
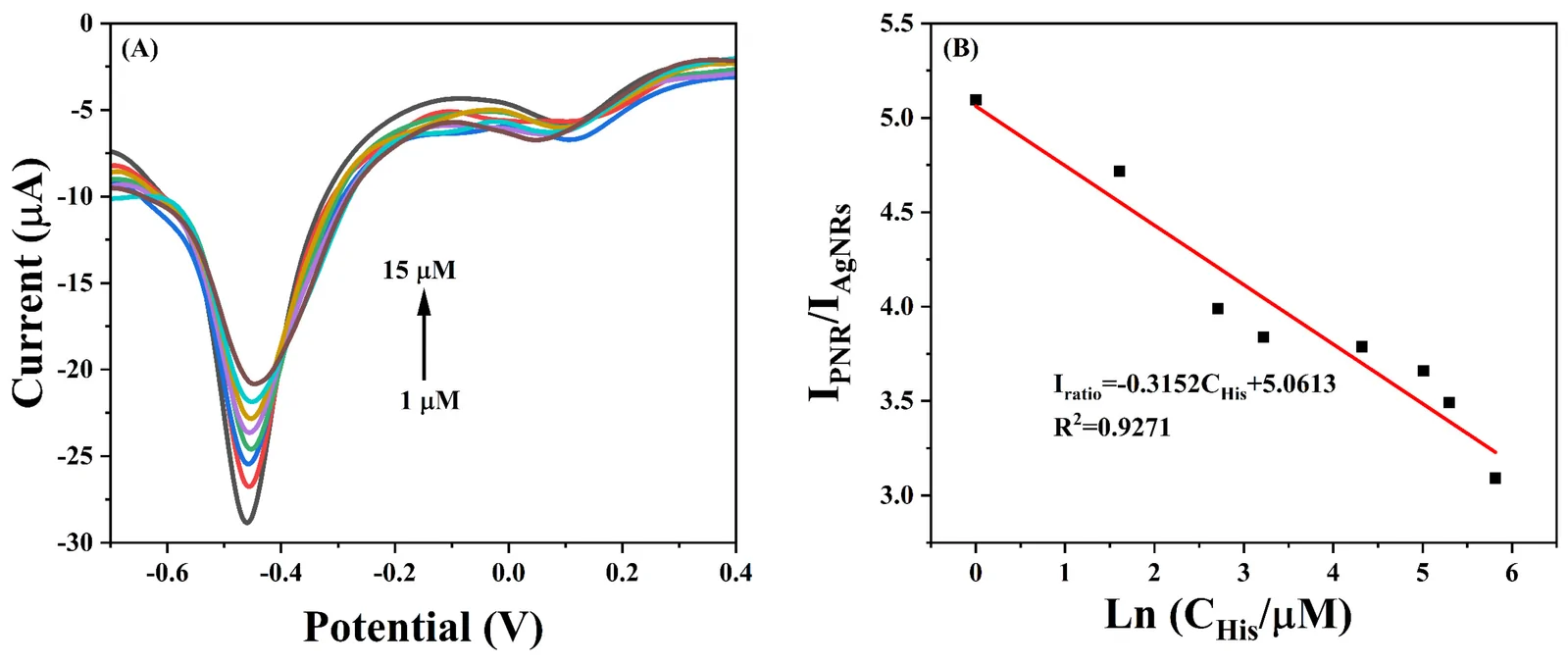
Ratiometric DPV curves of PNR-AgNRs-CNTs/GCE toward histamine. (**A**) DPV curves recorded in 0.1 M PBS (pH 6.0) upon successive additions of histamine (1–150 µmol/L); pulse amplitude: 50 mV, pulse width: 0.05 s, pulse period: 0.5 s. (**B**) Calibration plot of the I_PNR_/I_AgNRs_ ratio versus the natural logarithm of histamine concentration, exhibiting a linear relationship over the range 1–150 µmol/L with the regression equation.

Mechanism of ratiometric detection: Histamine molecules inherently lack direct electrochemical activity and cannot be directly detected by conventional electrochemical methods. In this study, the detection mechanism relies on the competitive inhibition effect of histamine on the PNR electrochemical signal. As illustrated in Figure 4A,B, when histamine (20 μmol/L) was introduced into the electrolyte, both anodic and cathodic peak currents decreased due to the non-conductive nature of histamine affecting solution conductivity. Notably, the current at the PNR characteristic peak (−0.41 V) decreased more significantly, which was attributed to the competitive interaction between histamine and PNR that inhibited the electrochemical reaction of PNR. In contrast, the AgNRs signal at approximately 0.1 V showed minimal change, as histamine did not significantly affect the silver redox process. This differential response forms the basis for ratiometric detection: the ratio of PNR to AgNRs current signals (I_PNR_/I_AgNRs_) can be used to indirectly quantify histamine concentration. To obtain direct experimental evidence for the interaction between histamine and the PNR-AgNRs-CNTs/GCE interface, electrochemical impedance spectroscopy (EIS) was performed in 0.1 M PBS (pH 6.0) before and after the addition of 50 µmol/L histamine (Figure S2). The Nyquist plot showed that the semicircle diameter, which reflects the interfacial charge-transfer resistance (*R*<sub>), remained essentially unchanged upon histamine addition, indicating that the electrode structure itself was not altered. By contrast, the slope of the low-frequency linear region, which is associated with mass-transfer and electron-transport kinetics between the electrode and the electrolyte, decreased slightly. This observation is consistent with the DPV results shown in Figure 5 and supports the proposed mechanism: histamine adsorbs onto the PNR layer via phosphate-mediated ion-pairing, thereby hindering electron transfer at the electrode–solution interface without disrupting the underlying AgNRs–CNTs conductive scaffold. We therefore qualify the detailed ion-pairing pathway as a proposed mechanistic hypothesis informed by the present EIS data and the prior literature on PNR–biogenic amine interactions, rather than as an unequivocally established explanation.

Optimization of experimental conditions: The experimental conditions were systematically optimized to maximize sensor performance. The optimal parameters were determined to be: electrolyte pH 6.0 (as shown in Figure 4B), adsorption time 6 min (as shown in Figure 4C), and CNTs material loading 0.08 mg/cm^2^ (as shown in Figure 4D). At pH 6.0, the sensor exhibited the most sensitive response to histamine with optimal signal stability. The 6 min adsorption time allowed sufficient interaction between histamine and the PNR layer without excessive background interference. The 0.08 mg/cm^2^ material loading provided adequate active sites while maintaining good electrode conductivity.

Analytical performance: Under the optimized conditions, DPV was employed for histamine detection. As shown in Figure 5A, as the histamine concentration increased from 1 to 150 μmol/L, the PNR characteristic peak current (I) exhibited a significant downward trend, while the AgNRs characteristic peak current (I) showed minimal variation. The selective attenuation of the PNR signal originates from phosphate-mediated ion-pair formation between protonated histamine and the cationic PNR backbone, which restricts polymer doping/de-doping dynamics, whereas the AgNRs redox signal remains invariant because histamine does not participate in silver oxidation/reduction or alter the nanorod surface chemistry [27]. The observed attenuation of the PNR electrochemical signal upon histamine addition can be rationalized by a phosphate-mediated ion-pairing mechanism, which was consistent with the previous literature. In PBS electrolyte, dihydrogen phosphate ions (H_2_PO_4_^−^) are rapidly adsorbed onto the cationic PNR scaffold via electrostatic attraction, forming an initial PNR–phosphate ion-pair complex [27]. Histamine exists predominantly as a dication (H_2_N^+^–R–NH_3_^+^) at acidic conditions. The positively charged histamine interacts with the PNR–phosphate complex through a second ion-pairing event, wherein the phosphate anion acts as a molecular bridge between the cationic PNR backbone and the protonated histamine. This ternary ion-pair assembly alters the local charge environment and restricts the doping/de-doping dynamics of the PNR film, manifesting as a decrease in redox peak current and a positive shift in formal potential. Accordingly, the *I_PNR_/I_AgNRs_* ratio was calculated and plotted against the natural logarithm of histamine concentration (ln C). As depicted in Figure 5B, a good linear relationship was obtained in the range of 1–150 μmol/L, with the linear equation: *I_PNR_/I_AgNRs_* = −0.3152 ln (*C_His_*) + 5.0613 (The unit of I_PNR_ and I_AgNRs_ is μA, while the unit of C_His_ is μmol/L).

The correlation coefficient R^2^ was 0.9271, and the detection limit (LOD) was calculated to be 0.16 μmol/L (S/N = 3). A comprehensive comparison with recently reported electrochemical histamine sensors [34,35,36,37,38] was presented in Table 1, highlighting that the proposed ratiometric strategy not only achieved a competitive detection limit (0.16 µmol/L) and linear range (1–150 µmol/L), but more importantly addressed the critical limitation of single-signal sensors—their inherent susceptibility to matrix interference and instrumental drift-through an intrinsic self-referencing dual-signal mechanism. The results revealed that the as-fabricated sensor featured a superior detection limit relative to previously reported electrodes, with no sacrifice in linear range. Moreover, unlike the single-signal strategies employed by the other sensors listed in Table 1, the proposed ratiometric approach leverages dual electrochemical responses—one sensitive to histamine and the other serving as an internal reference—to inherently compensate for matrix interference and instrumental drift. In contrast to the single-signal electrochemical sensors summarized in Table 1 (especially Ag-Ag_2_O/CNTs/GCE) [37], the developed ratiometric sensor utilized a dual-response mechanism that functions as a built-in internal reference. This feature effectively suppressed matrix-induced errors without requiring additional calibration or complex sample pretreatment.

It is important to note that the observed current attenuation upon histamine addition is not attributable to a histamine-specific molecular recognition event. Rather, as demonstrated by Kumar [27] for cadaverine and putrescine, the sensing mechanism relies on phosphate-mediated ion-pair formation between protonated polyamines and the cationic PNR backbone. Consequently, any biogenic amine that exists in a multiply protonated state under the experimental pH (e.g., putrescine, cadaverine, tyramine, and spermidine) would be expected to interact with PNR through the same ion-pairing pathway and generate a comparable electrochemical response. This intrinsic broad-spectrum responsiveness toward protonated biogenic amines is a characteristic feature of the PNR recognition element, not an artifact of electrode fouling.

### 3.4. Selectivity, Stability, and Reproducibility

**Figure 6:**
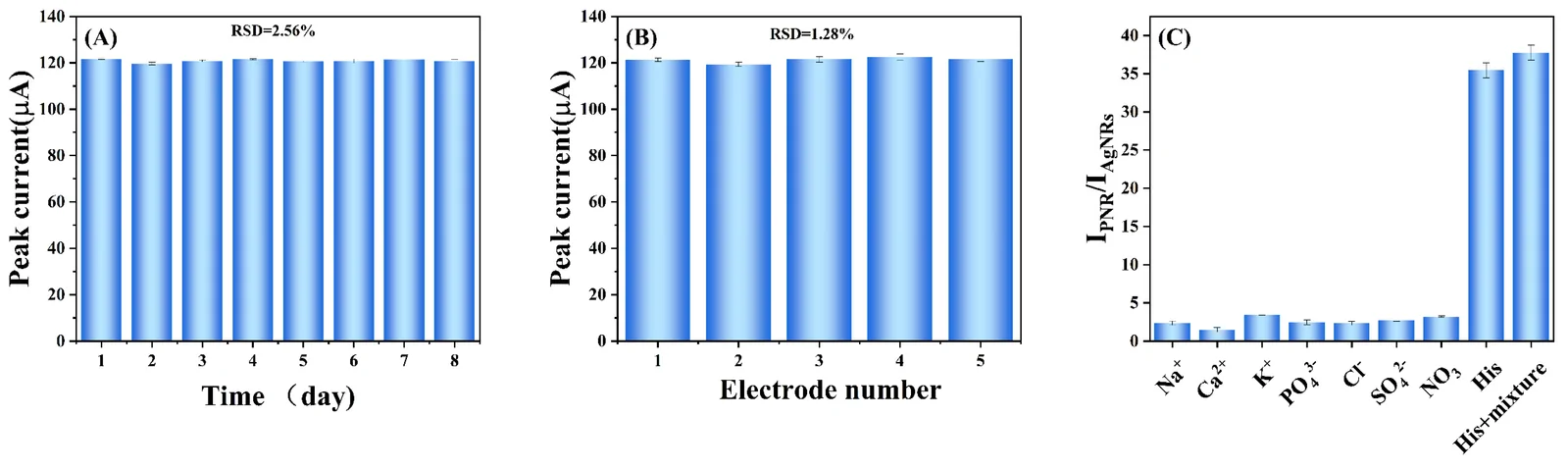
Stability (**A**), reproducibility (**B**) and selectivity (**C**) performances for the obtained electrode in electrochemical determination of histamine. All the measurements were performed in 0.1 M KCl solution as the electrolyte.

Selectivity: The anti-interference capability of the sensor was evaluated by testing the response toward common interfering substances including Na^+^, Ca^2+^, K^+^, PO_4_^3−^, and NO_3_^−^ at concentrations of 1.5 mM, as well as a mixture of these ions. As shown in Figure 6C, the presence of these common interfering ions, either individually or in combination, did not significantly affect the *I_PNR_/I_AgNRs_* ratio for histamine detection (variation < 5%), demonstrating excellent selectivity and anti-interference ability of the proposed sensor. To further evaluate the sensor’s response toward co-occurring biogenic amines in spoiled aquatic products, putrescine, cadaverine, and spermidine were tested at 150 µmol/L under the optimized conditions. As shown in Figure S1 (Supplementary Materials), all three amines produced a response profile analogous to that of histamine: the AgNRs signal remained essentially invariant, whereas the PNR characteristic peak current decreased significantly, resulting in altered currents ratios. These results confirm that the phosphate-mediated ion-pairing mechanism is broadly applicable to protonated polyamines, consistent with previous reports on PNR sensors [27,28]. While this cross-reactivity precludes histamine-specific discrimination, it reinforces the utility of the proposed platform as an electrochemical proxy for total biogenic amine burden in freshness assessment.

Stability: The long-term stability of the sensor was assessed by continuous detection over 8 days using the same PNR-AgNRs-CNTs/GCE electrode under identical conditions (pH 6.0, histamine concentration 20 μmol/L). As shown in Figure 6A, the average peak current was 120.57 ± 3.09 μA with an RSD of 2.56%. The existing data demonstrate minimal current drift (RSD = 2.56%) and negligible signal attenuation over the 8-day period, suggesting that the PNR-AgNRs-CNTs film maintains robust electrochemical integrity under refrigerated storage. We acknowledge that the 8-day stability assessment represents an initial evaluation under laboratory storage conditions, and we note that extended long-term studies (e.g., 30–60 days) are warranted in future work to fully establish the sensor’s shelf life for routine field deployment.

Reproducibility: The reproducibility was evaluated by preparing eight PNR-AgNRs-CNTs/GCE electrodes under identical conditions and testing their response to 20 μmol/L histamine. As shown in Figure 6B, the average peak current was 121.52 ± 1.56 μA with an RSD of 1.28%, demonstrating excellent batch-to-batch reproducibility of the electrode fabrication protocol.

Since the detection mechanism is the competitive inhibition of PNR by biogenic amines (including histamine, putrescine, cadaverine, and tyramine), the obtained electrochemical sensors used are unable to distinguish between various individual biogenic amines, such as putrescine, cadaverine and spermidine. However, during the spoilage of aquatic products, histamine, putrescine, cadaverine, and tyramine are produced synergistically by microbial decarboxylation rather than in isolation. Regulatory frameworks such as the EU standard (EC) No. 2073/2005 and China’s GB 2733-2015 set limits not only for histamine but also implicitly recognize the cumulative toxicity of biogenic amines. In this regard, the PNR-based ratiometric sensor functions analogously to the total volatile basic nitrogen (TVB-N) assay—a well-established bulk parameter for freshness—by responding to the collective biogenic amine load rather than to histamine alone. Therefore, the sensor is better positioned as a rapid screening tool for overall spoilage degree, using histamine as a model and calibration reference, rather than as a histamine-specific detector.

### 3.5. Real Sample Analysis

The practical applicability of the developed sensor was demonstrated by detecting histamine in crayfish samples. Three different crayfish samples were analyzed using the standard addition method at three concentration levels (1, 5, and 10 μmol/L). For each batch of crayfish samples, a random sample was selected and repeated tests were conducted (*n* = 7). Prior to spiking experiments, the native histamine concentration in each untreated crayfish homogenate was determined independently using the proposed electrochemical sensor and the standard method (GB 5009.208-2016). The background concentrations were found to be 0.82 ± 0.15 µmol/L for Crayfish sample 1, 1.24 ± 0.21 µmol/L for Crayfish sample 2, and 0.95 ± 0.18 µmol/L for Crayfish sample 3, respectively. These values were used as C_background_ in the recovery calculation according to the equation given in Section 2.8. The calculated recoveries ranged from 95.9% to 102.3% (Table 2). Moreover, compared with the standard method (referred to as GB 5009.208-2016), the RSD values were presented as 1.23–2.64%, indicating high accuracy and precision of the method for real sample analysis. The relatively large standard deviations in the electrochemical detection amount column reflect inherent spatial heterogeneity of biogenic amine distribution in crayfish tissue (e.g., between outer and inner muscle), rather than methodological imprecision; this is corroborated by comparable variations in the standard method results. A paired *t*-test of the corresponding data from both methods yielded *p* > 0.05, confirming that there was no statistically significant difference between the proposed electrochemical sensor and the national standard method (GB 5009.208-2016).

The proposed platform offers substantial practical advantages for routine monitoring: electrode fabrication employs low-cost, commercially available materials via simple drop-casting and electropolymerization, while the sample pretreatment is markedly simpler than chromatographic alternatives, positioning the method as a cost-effective, field-deployable screening tool.

## 4. Conclusions

This study demonstrates that a ratiometric electrochemical strategy based on PNR-AgNRs/CNTs composites enables reliable quantification of histamine in complex biological matrices without requiring its direct electrochemical oxidation. By exploiting the competitive inhibition of histamine toward the PNR redox process while employing the AgNRs response as an internal reference, the *I_PNR_*-*I_AgNRs_* dual-signal approach intrinsically compensates for electrode-to-electrode variability, instrument drift, and matrix-induced signal fluctuations—limitations that compromise the reliability of conventional single-signal sensors. The close agreement between the sensor and the national standard method (GB 5009.208-2016) in crayfish samples confirms that the proposed platform achieves sufficient accuracy and precision for practical food safety monitoring. These findings suggest that ratiometric electrochemical sensing offers a robust and field-deployable solution for on-site histamine screening in aquatic products, with potential to streamline freshness assessment and quality control workflows in seafood supply chains.

## Figures and Tables

**Table 1:** Comparison of His detected by different electrochemical methods.

No.	Electrodes	Linear Range (μmol/L)	LOD (μmol/L)	Ref.
1	Unmodified SPGE	5–100	0.62	[34]
2	Nafion-CNTs/GCE	20–200	0.39	[35]
3	Au/Cys/MIP	0.5–1000	0.21	[36]
4	MIP//AuNPs/GCE	1–107	0.6	[37]
5	Ag-Ag_2_O/CNTs/GCE	5–200	0.18	[22]
6	SWCNT/CPE	4–720	1.26	[38]
7	PNR-AgNRs-CNTs/GCE	1–150	0.16	This work

**Table 2:** Results of recovery rate measurement for real crayfish samples (*n* = 7).

Samples	Adding Amount (μmol/L)	Detected Amount (μmol/L)	Recovery (%)	Detected Amount by Standard Method (μmol/L)	RSD with Standard Method (%)
Crayfish sample 1	1	1.15 ± 1.01	95.75	1.03 ± 1.15	1.23
	5	5.37 ± 1.28	96.39	5.24 ± 1.11	2.15
	10	10.2 ± 2.09	99.27	10.19 ± 2.21	1.77
Crayfish sample 2	1	1.49 ± 1.96	101.16	1.27 ± 1.06	2.53
	5	5.24 ± 1.32	96.84	5.28 ± 1.27	1.7
	10	10.0 ± 2.28	99.37	10.25 ± 2.14	2.03
Crayfish sample 3	1	1.36 ± 1.85	97.92	0.97 ± 1.58	2.15
	5	5.14 ± 1.53	98.41	4.96 ± 1.33	2.64
	10	10.9 ± 2.06	99.28	10.01 ± 2.19	2.23

## Data Availability

The original contributions presented in this study are included in the article/Supplementary Materials. Further inquiries can be directed to the corresponding authors.

## Supplementary Materials

The following supporting information can be downloaded at: https://www.mdpi.com/article/10.3390/foods15162917/s1, Scheme S1. Formation of ion pair complex between neutral red and dihydrogen phosphate ions (step 1) and interaction of putrescine/cadaverine with the ion-pair complex (step 2) (Ref. [27]).; Figure S1. DPV curves of the PNR-AgNRs-CNTs/GCE recorded in 0.1 M PBS (pH 6.0) upon addition of 150 µmol/L histamine (HA), putrescine (Put), cadaverine (Cad), and spermidine (Spm). Pulse amplitude: 50 mV; pulse width: 0.05 s; pulse period: 0.5 s. Figure S2. EIS curves of the PNR-AgNRs-CNTs/GCE recorded in 0.1 mol/L PBS electrolyte (black line) and in 0.1 mol/L PBS + 150 µmol/L His electrolyte (red line).

## Abbreviations

The following abbreviations are used in this manuscript:

Abbreviations	Full name
PNR	Poly Neutral Red
NRs	Nanorods
GCE	Glassy carbon electrode
DPV	Differential Pulse Voltammetry
CNTs	Carbon nanotubes
XRD	X-ray diffraction
FTIR	Fourier transform infrared spectroscopy
SEM	Scanning electron microscopy
TGA	Thermogravimetric analysis
CV	Cyclic voltammetry
His	Histamine
Cad	Cadaverine
Put	Putrescine
Spm	Spermine
