# Supplementary material for: Poly(Neutral Red)–Silver Nanorods–Carbon Nanotubes Composite-Based Ratiometric Electrochemical Sensor for Rapid Detection of Histamine in Crayfish
Source: Foods. 2026 Aug 20;15(16):2917. doi: 10.3390/foods15162917 (PMC13512178; doi:10.3390/foods15162917)
Supplement: Supplementary file 1 [file foods-15-02917-s001.zip › foods-4418079-supplementary.pdf]

Section S1

2. Materials and Methods

2.1. Reagents and materials

Silver nitrate ($AgNO_3$, analytical grade), 2-mercaptobenzoic acid (2-MBA, analytical grade), ammonia solution (25%, analytical grade), carbon nanotubes (CNTs, purity > 95%), neutral red (analytical grade), sodium nitrate ($NaNO_3$, analytical grade), Nafion solution (5% wt), isopropanol (analytical grade), potassium dihydrogen phosphate, and disodium hydrogen phosphate (analytical grade, for PBS buffer preparation) were purchased from standard chemical suppliers. Histamine standard (obtained from Sigma-Aldrich, purity ≥ 98%) was used for calibration. Potassium ferricyanide ($K_3[Fe(CN)_6]$, analytical grade) and potassium chloride (KCl, analytical grade) were used for electrochemical characterization. Trichloroacetic acid (analytical grade), sodium hydroxide (analytical grade), and other reagents were of analytical grade. Ultrapure water (18.2 MΩ·cm) was used throughout all experiments.

2.2. Instrumentation

Electrochemical measurements were performed on a CHI760E electrochemical workstation (Chenhua Instrument, Shanghai, China) with a conventional three-electrode system: a modified glassy carbon electrode (GCE, diameter 3 mm) as the working electrode, a platinum wire as the counter electrode, and an Ag/AgCl (saturated KCl) electrode as the reference electrode. Scanning electron microscopy (SEM) with energy-dispersive X-ray spectroscopy (EDS) was employed for surface morphology and elemental analysis. X-ray diffraction (XRD) was conducted using an X-ray diffractometer. Raman spectroscopy was performed on a laser confocal micro-Raman system (532 nm laser, 10 mW power). Fourier transform infrared spectroscopy (FTIR) and thermogravimetric analysis (TGA) were used for compositional and thermal stability characterization. A UV-visible spectrophotometer (Yuanxi Instrument, Shanghai, China) was used for the national standard method validation.

2.3. Synthesis of silver nanorods (AgNRs)

AgNRs were synthesized via a liquid-phase reduction method with optimization based on literature procedures [27]. Briefly, 155 mg of 2-MBA and 170 mg of $AgNO_3$ were co-dissolved in 6 mL of deionized water and ultrasonicated for 10 min to ensure complete dissolution. Under continuous ultrasonication, 0.5 mL of concentrated ammonia solution (25%) was added dropwise at approximately 0.1 mL/min. The reaction continued for 20 min under ultrasonication until the solution turned into a yellow transparent dispersion that did not fade within 30 s, indicating successful formation of AgNRs. The resulting AgNRs dispersion was transferred to a centrifuge tube and stored at 4 °C for subsequent use.

2.4. Fabrication of PNR-AgNRs-CNTs/GCE

Pretreatment of GCE: The glassy carbon electrode (diameter 3 mm) was sequentially polished on a microcloth with 0.3 μm and 0.05 μm alumina slurry in a figure-8 pattern until a mirror-like surface was achieved. The electrode was thoroughly rinsed with ultrapure water, then sequentially sonicated in nitric acid (1:1, v/v), acetone, and absolute ethanol for 2 min each, followed by rinsing with ultrapure water and drying under nitrogen flow.

Preparation of AgNRs-CNTs/GCE: An accurately weighed amount of CNTs (1 mg) was placed in a 1.5 mL centrifuge tube, followed by sequential addition of 1 mL isopropanol, 100 μL of freshly synthesized AgNRs dispersion, and 10 μL of 5% Nafion solution. The mixture

was vortexed for 2 min and then ultrasonicated for 10 min to obtain a uniform AgNRs-CNTs suspension. The pretreated GCE was modified by dropping 2 μL of the suspension four times (8 μL total) onto the electrode surface, with each drop dried under an infrared lamp for 5 min before the next application. After complete drying, the AgNRs-CNTs/GCE was obtained.

Preparation of PNR-AgNRs-CNTs/GCE: A neutral red polymerization solution was prepared by dissolving 2.3 mg of neutral red in 20 mL of 0.1 M phosphate-buffer saline (PBS, pH 6.0) containing 0.1 M $NaNO_3$, followed by 5 min ultrasonication to form a stable magenta solution. The AgNRs-CNTs/GCE was immersed in this solution, and cyclic voltammetry was performed in the potential range of −0.8 to 0.8 V at a scan rate of 50 mV/s for 20 cycles. During the scanning process, oxidation and reduction peaks of neutral red appeared at approximately −0.41 V and −0.36 V, respectively, indicating progressive electrochemical polymerization on the electrode surface. When the current curve gradually stabilized (after approximately 15 cycles), it indicated the formation of a stable polymer film. After polymerization, the electrode was rinsed with ultrapure water three times to remove residual neutral red monomers and dried at room temperature. The resulting PNR-AgNRs-CNTs/GCE was stored at 4 °C for subsequent use.

2.5. Sample pretreatment

Crayfish samples were purchased from local markets. The pretreatment procedure was performed according to the standard T/QJCIPA 002-2023 "Quality Requirements for Frozen Crayfish Tails" with modifications [27]. Live crayfish were euthanized, and the heads, tails, and shells were removed. The muscle tissue was collected and homogenized using a homogenizer. Accurately weighed 5.0 g of the homogenized sample was transferred to a 50 mL centrifuge tube, and 20 mL of 10% trichloroacetic acid (TCA) solution was added. The mixture was thoroughly vortexed, then transferred to a 55 °C water bath for 10 min, followed by ultrasonic extraction for 20 min (with manual shaking every 5 min). The extract was filtered through filter paper, and the residue was washed with deionized water three times. The filtrates were combined, and the pH was adjusted to neutral using 250 g/L sodium hydroxide solution. The solution was transferred to a 100 mL volumetric flask and diluted to volume. The prepared sample solution was stored at 4 °C and analyzed within 24 h.

2.6. Electrochemical measurements

All electrochemical measurements were conducted in 0.1 M phosphate buffered saline (PBS, the pH value is 6.0) at room temperature. The electrochemical techniques employed included:

Cyclic voltammetry (CV): Potential window −0.8 to 0.8 V, scan rate 50–300 mV/s, used for electrode performance evaluation and PNR polymerization monitoring.

Differential pulse voltammetry (DPV): Potential window −0.8 to 0.6 V, pulse amplitude 50 mV, pulse width 0.05 s, pulse period 0.5 s, quiet time 2 s, used for histamine quantitative detection.

Electrochemical impedance spectroscopy (EIS): Frequency range 0.1 Hz to 100 kHz, amplitude 5 mV, in 5 mmol/L $K_3[Fe(CN)_6]$/0.1 M KCl solution, used for charge transfer resistance evaluation.

Chronocoulometry (CC): Potential step to 0.5 V, sampling interval 0.1 s, in 5 mmol/L $K_3[Fe(CN)_6]$ solution, used for electrochemically active surface area determination.

2.7. National standard method validation

To validate the accuracy of the proposed electrochemical method, the national standard method (GB 5009.208-2016) was employed as a reference [28]. Briefly, 10 g of crayfish sample

was extracted with 20 mL of 10% TCA solution by oscillation for 2 min. The extract was filtered, and 3 mL of n-pentanol was added for liquid-liquid extraction (5 min shaking, repeated three times). The combined organic phase was back-extracted with 3 mL of hydrochloric acid solution (1:1, v/v) three times. The combined aqueous phase (2 mL) was transferred to a 10 mL colorimetric tube, and the absorbance was measured at 480 nm using a UV-visible spectrophotometer after azo-coupling reaction. The histamine concentration was quantified by the standard curve method.

2.8. Data analysis

The recovery and relative standard deviation (RSD) were calculated according to the following equations:

$$\text{Recovery (\%)} = (C_{detected} - C_{background})/Cspiked \times 100\%$$

$$\text{RSD(\%)} = SD/Mean \times 100\%$$

All measurements were performed at least in triplicate, and data were expressed as mean ± standard deviation.

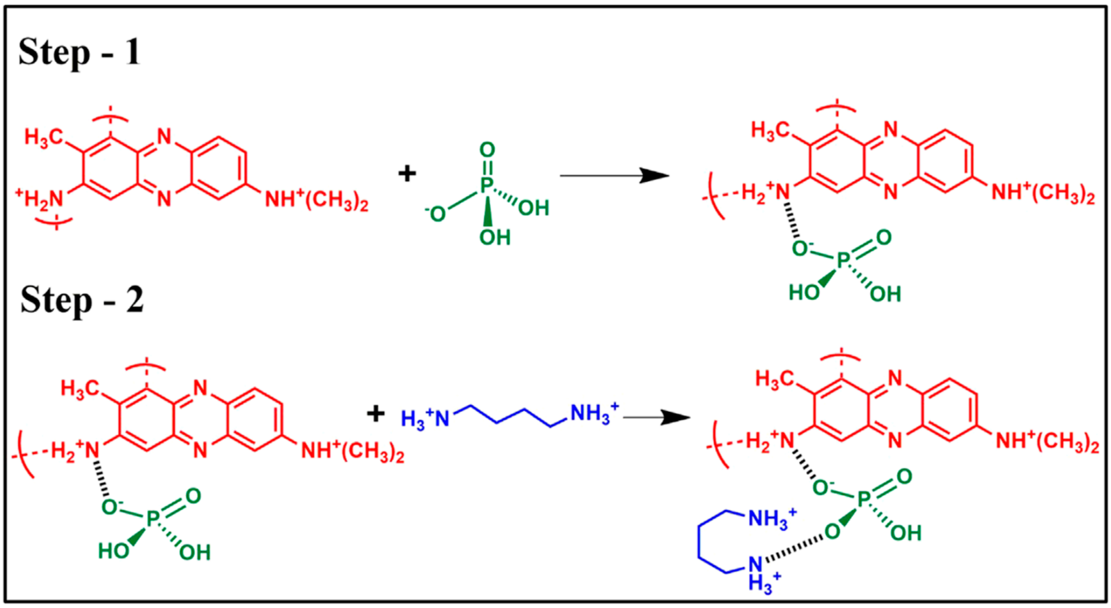

Scheme S1. Formation of ion pair complex between neutral red and dihydrogen phosphate ions (step 1) and interaction of putrescine/cadaverine with the ion-pair complex (step 2) (Ref. [27]).

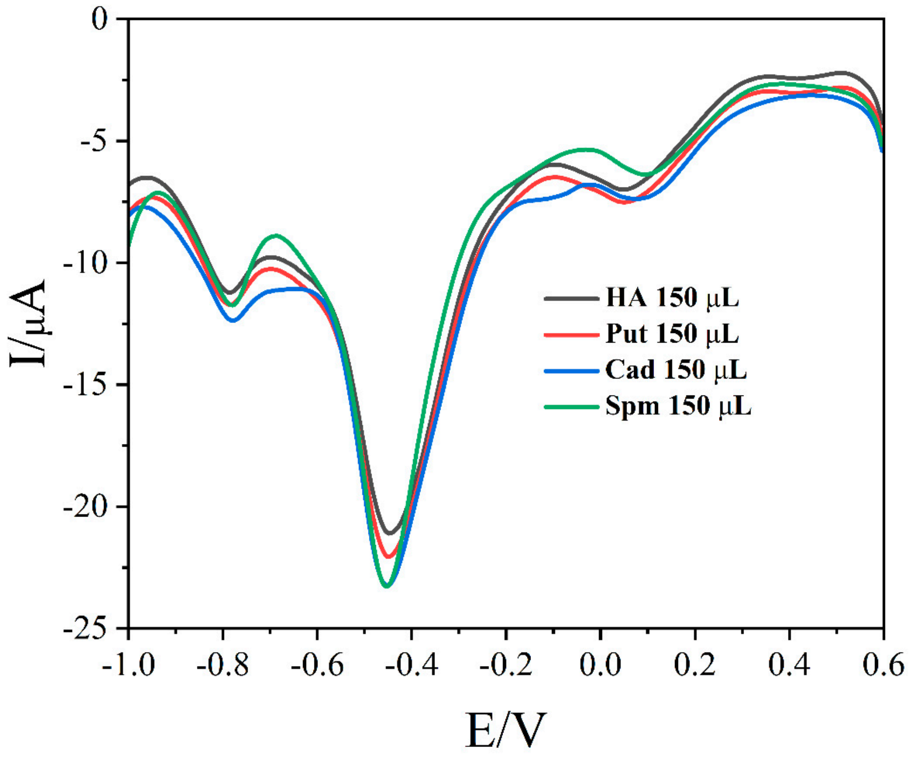

Figure S1. DPV curves of the PNR-AgNRs-CNTs/GCE recorded in 0.1 M PBS (pH 6.0) upon addition of 150 µmol/L histamine (HA), putrescine (Put), cadaverine (Cad), and spermidine (Spm). Pulse amplitude: 50 mV; pulse width: 0.05 s; pulse period: 0.5 s.

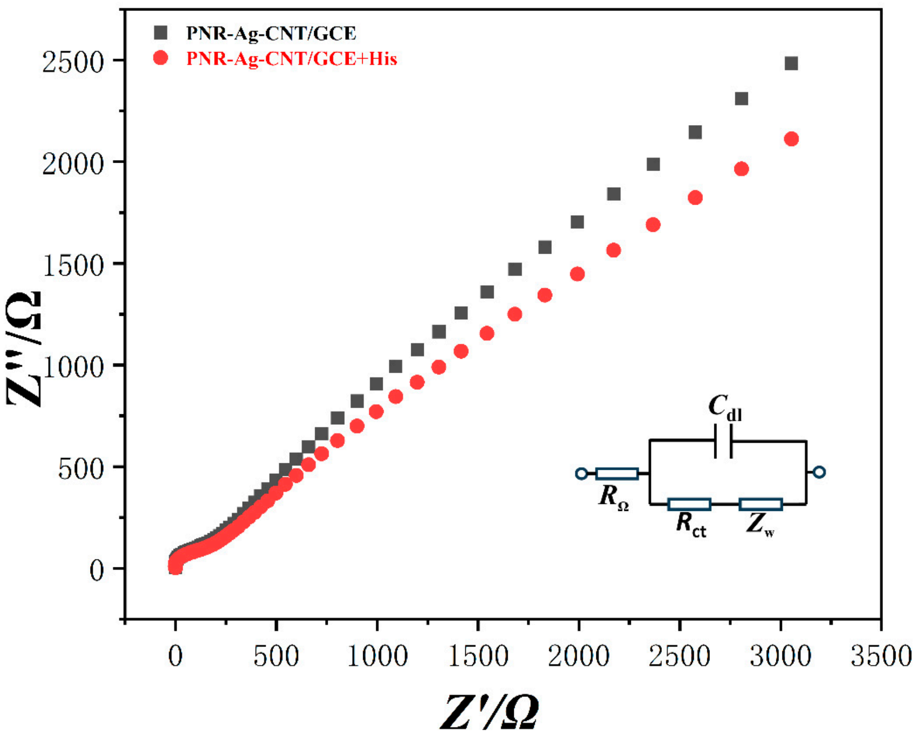

Figure S2. EIS curves of the PNR-AgNRs-CNTs/GCE recorded in 0.1 mol/L PBS electrolyte (black line) and in 0.1 mol/L PBS + 150 μmol/L His electrolyte (red line).
